# Effect of Rumen Protected Methionine and α-Tocopherol on Growth Performance, Carcass Characteristics, and Meat Composition of Late Fattening Hanwoo Steer in High-Temperature Seasons

**DOI:** 10.3390/ani10122430

**Published:** 2020-12-18

**Authors:** Byung-Ki Park, Jun-Sang Ahn, Min-Ji Kim, Gi-Hwal Son, Sang-Hun Bong, Deok-Yun Gil, Joong-Kook Park, Chang-Woo Lee, Eung-Gi Kwon, Sun-Sik Chang, Jong-Suh Shin

**Affiliations:** 1 Department of Animal Science, Kangwon National University, Chunchoen 24341, Korea; animalpark@hanmail.net (B.-K.P.); bambingu08@naver.com (M.-J.K.); oscar0202@naver.com (G.-H.S.); snsdy616@daum.net (D.-Y.G.); 2 Hanwoo Research Institute, National Institute of Animal Science, RDA, Pyeongchang 25340, Korea; dkswns121@korea.kr (J.-S.A.); kug2237@korea.kr (E.-G.K.); jangsc@korea.kr (S.-S.C.); 3 NUBO Bio & Technologies, Seoul 01838, Korea; bbongsh@daum.net; 4 Nonghyup Feed Co., Ltd., Seoul 05398, Korea; jkpark203@hanmail.net; 5 Kangwon Livestock Technology Research Institute, Hoengseong 25266, Korea; k97112603@korea.kr

**Keywords:** carcass characteristics, Hanwoo steers, meat composition, methionine, α-tocopherol

## Abstract

**Simple Summary:**

Over the past 100 years, the average temperature in Korea has risen by 1.8 ℃, more than double the world average (0.75 ℃). High temperatures in summer can cause a decrease in feed intake, body weight gain, rumen mobility, and liver function. This study was conducted to investigate the effects of rumen-protected methionine and α-tocopherol (RPMT) on the growth performance, carcass characteristics, and meat composition of late fattening Hanwoo steers in high-temperature seasons. In this study, supplementations with RPMT are considered to be beneficial to average daily gain, feed conversion ratio, and Commission Internationale de l’Eclairage color stability without adversely affecting the feed intake of the late fattening Hanwoo steers in high-temperature seasons. Thus, supplementations of RPMT has desirable effects on the performance traits in beef cattle and prevents oxidation of longissimus muscle. Henceforward, further studies with more animals and increase the mounts of addition are needed.

**Abstract:**

This study was conducted to investigate the effects of rumen-protected methionine and α-tocopherol (RPMT) on growth performance, carcass characteristics, and meat composition of late fattening Hanwoo steers in high-temperature seasons. Fourteen steers were randomly assigned to one of two diets; control (commercial concentrate) and treatment (commercial concentrate + 20 g of RPMT). Average daily gain was 34% higher in the treatment group than in the control group; however, there was no significant difference due to the small number under investigation. Feed conversion ratio was lower in the treatment group than in the control group (*p* < 0.05). Supplementation of RPMT had little effect on the fatty acid composition of longissimus muscle. Metmyoglobin in the longissimus muscle was significantly lower in treatment group compared to the control group at the ninth day of storage (*p* < 0.05). The redness of the longissimus muscle was higher in the treatment group than in the control group on day 9 of storage (*p* < 0.01). Thus, the results suggest that RPMT have positive effects on growth performance, and Commission Internationale de l’Eclairage color stability in the longissimus muscle of late fattening Hanwoo steers in high-temperature seasons.

## 1. Introduction

Over the past 100 years, the average temperature in Korea has risen by 1.8 ℃, more than double the world average (0.75 ℃), and is expected to rise by 3.2 °C in 2050. In addition, it is expected that the periods of heat waves and tropical nights will increase, and that most areas except the inland regions will be subtropical [1]. High temperatures in summer can cause a decrease in feed intake, body weight gain, rumen mobility, and liver function. In the late fattening period, which is accompanied by a decrease in ruminal and liver function due to long-term high energy diet feeding, high temperature stress is likely to decrease quality and yield grade of carcass due to decreased feed intake, body weight gain, and energy efficiency. In order to solve these problems, some studies have been carried out in the fields of facility environment [2] and feed nutrition [3].

In recent years, there has been an increased interest in functional materials that can improve the growth performance and meat quality (marbling score and meat color, etc.) of late fattening Hanwoo steers. Methionine, a limiting amino acid in cattle [4], is considered to improve growth performance and meat quality of beef cattle in summer. Methionine acts as the initiation codon for methylation and protein synthesis necessary for cell growth and metabolism [5], and also participates in fat metabolism for intramuscular fat synthesis [6]. In addition, methionine has been reported to improve liver function by degrading triglycerides in the liver [7].

In the late fattening period, risk of exposure to oxidative conditions is high due to long-term fattening and high-temperature stress [8]. At this time, α-tocopherol, the most active of vitamin E compounds, can act as a powerful antioxidant in beef cattle. α-tocopherol has been known to prevent oxidation of cell membranes, muscle fibers, and fats [9] and increase the safety of meat color [10] and improve feed efficiency [11]. However, to date, studies on methionine and α-tocopherol have been performed independently in cattle, and studies on cattle under high temperature conditions are rare. In order to improve the supplement effects in beef cattle, a coating processing is essential to control rumen degradation [12]. However, there are no related studies in Hanwoo steer (Korean native cattle).

Therefore, this study was conducted to investigate the effects of rumen-protected methionine and α-tocopherol on the growth performance, carcass characteristics, and meat composition of late fattening Hanwoo steers in high-temperature seasons.

## 2. Materials and Methods

### 2.1. Animals, Treatments, and Management

This study was conducted using fourteen Hanwoo steers (713.3 ± 85.2 kg, castration age: 14.2 ± 0.9 months old) at the Institute of Livestock Technology Research from June to October, 2017. During this period, the average temperature was 23.5 °C (Max: 34.20 °C, Min: 9.0 °C), humidity was 89.7 (Max: 99.9%, Min: 40.0%), and Temperature Humidity Index (Formula: 0.8 × temperature + humidity/100 × (temperature − 14.4) + 46.4) was 73.19 (Max: 88.7, Min: 54.9). Protocols involving the use of experimental animals were approved by the ethical and scientific guidelines of the Animal Experiment Ethics Committee of Kangwon National University (No: KIACUC-16-0010).

Fourteen Hanwoo steers were randomly assigned to one of two dietary treatments based on a completely randomized block design. The control group was fed commercial concentrate + rice straw, and the treatment group was fed commercial concentrate + rice straw + 20 g (steer/day) of RPMT. RPMT used in present study comprised 15% methionine + 0.5% α-tocopherol (1.1 IU/mg) + 84.5% rumen protected fat, and the fat used in coating the methionine and α-tocopherol was manufactured by a mixing reaction of palm oil and calcium.

The commercial concentrate (5 kg) and rice straw (0.75 kg) were fed twice daily (08:00, and 17:00). RPMT was mixed and fed when feeding concentrated feed during the test period (June to October, 2017). Water was always freely available, and other feeding management was conducted in accordance with the practices of the Institute of Livestock Technology Research. The chemical compositions of the experimental diets are presented in Table 1.

### 2.2. Growth Performance

Feed intake and residual quantity was measured daily before morning feeding for calculate the actual intake of feed. Average daily gain (ADG) was calculated based on the number of days every month, and dry matter intake (DMI) and ADG were used for calculating Feed conversion ratio (FCR). The chemical compositions of the experimental diets were analyzed using the standard methods of the AOAC [13]. Neutral detergent fiber (NDF) and acid detergent fiber (ADF) was analyzed by the methods of Van Soest et al. [14] using a filter bag (Ankom F57, Ankom Technology, Macedon, NY, USA).

### 2.3. Carcass Characteristics

All animals were slaughtered to assess carcass yield (carcass weight, backfat thickness, and rib eye area) and quality grades (marbling score, meat color, fat color, texture, and maturity) at the local slaughterhouse. Carcass traits were determined at the 13th rib section from the left side of each carcass. Meat graders evaluate the carcass grade according to criteria of the Korean carcass grading system [15].

### 2.4. Pretreatment and Storage Test

The inedible fat and connective tissues of samples were removed in a low temperature room (5 °C) for analysis of chemical composition and storage tests. For storage testing, control, and treatment samples were cut into 1 cm pieces, and 12 pieces were prepared for each treatment group. The sample pieces were put in a polyethylene bag and stored at 4 °C for 9 days, and the quality characteristics according to the storage period were analyzed by using 3 pieces at intervals of 3 days from 0 days.

### 2.5. Chemical Composition and Quality of the Longissimus Muscle

The chemical compositions of the longissimus muscle were measured according to the method of AOAC [13]. Water holding capacity (WHC) was measured by the procedure described by Hofmann and White [16]. Briefly, a 0.3 g sample of muscle was placed in filter (Whatman No.1 GE Healthcare, Amersham, UK) press device and compressed for 5 min. After this process, WHC was calculated from duplicate samples as a ratio of the meat film area to the total area by a digitizing area-line meter (Super PLANIX-a, Tamaya Technics Inc., Tokyo, Japan).

Texture profile analysis (TPA) measurements were made by placing samples in a polyethylene bag and heating them in a water bath until the core temperature reached 75 °C. After cutting each longissimus muscle sample to 1 × 1 × 1 mm, the hardness, elasticity, cohesiveness, gumminess, and chewiness were measured using a texture analyzer equipped with a cylindrical probe of 35 mm diameter (Stable Micro Systems Co., Ltd., Godalming, UK). The samples were measured by pressing 80% of the sample height twice with a pretest, test, and posttest speed of 1 mm/s.

Fatty acid composition of the longissimus muscle was measured according to the methods of Folch et al. [17]. In brief, 0.5 g lyophilized samples were homogenized in chloroform–methanol (2:1) and 0.88% NaCl solution. After homogenizing, the bottom layer separated by centrifugation (1250 × g, 4 °C, 30 min) was transferred to another tube, and the organic solvent was flushed with nitrogen gas. Next, 1 mL of 0.5 *n* methanolic NaOH was added to the tube, the mixture was heated for 15 min, and then cooled. Two milliliters of 14% BF3-methanol were added, heated, and then cooled. After cooling, 1 mL heptane and 2 mL saturated NaCl solution was added, and the mixture was allowed to stand at room temperature for 40 min. The supernatant was transferred to a vial using a micropipette, and fatty acids were analyzed by gas chromatography (Shimadzu-17A, Shimadzu, Kyoto, Japan).

Deoxymyoglobin (DeoxyMb), oxymyoglobin (OxyMb), and metmyoglobin (MetMb) were measured according to the methods of Krzywicki [18]. The samples were packed in low density polyethylene wrap (Oxygen transmission rate: 35,273 cc /m^2^·24 h·atm, 0.01 mm thickness, 3M Co, Korea) for measurement. The reflectance was measured at 473, 525, 572, and 730 nm using a UV spectrometer (UV-240 1PC, Shimadzu Corp, Kyoto, Japan) and the percentage (%) of MetMb was calculated according to the method of Demos et al. [19]. R630 and R580, an indicator of red intensity by OxyMb, was calculated as the reflectance difference at 630 nm and 580 nm; DeoxyMb was calculated as 100 minus OxyMb and MetMb.

Commission Internationale de l’Eclairage (CIE) color was measured by colormeter (Colormeter CR-300, Minolta Co., Osaka, Japan). L* (lightness), a* (redness), and b* (Yellowness) values were measured repeatedly in the same manner.

For measurement of pH, approximately 10 g of *longissimus* muscle was cut into small pieces and homogenized with 90 mL of distilled water (PolyTron PT-2500 E, Kinematica, Lucerne, Switzerland). pH values were measured immediately after homogenization using a pH meter (Orion 230A, Thermo Fisher Scientific Inc., Miami, FL, USA).

The 2-thiobarbituric acid reactive substances (TBARS) values of the *longissimus* muscle were determined according to the method of Witte et al. [20]. Each sample (10 g) was added to 20% trichloro-acetic acid (in 2 M phosphoric acid) at 25 mL and homogenized with a homogenizer for 30 s. Samples were diluted at distilled water until amount of the homogeneous solution reached to 50 mL and then centrifuged (1250 × g, 4 °C, 10 min). After centrifugation, the supernatant was filtered using a filter paper. Next, 0.005 mM 2-thiobarbituric acid (5 mL) was added to the filtrate (5 mL), allowed to stand at room temperature for 15 h, and measured at 530 nm using a UV/VIS spectrophotometer (Molecular Device, M2e, Sunnyvale, CA, USA).

TBARS (mg of malondialdehyde ÷ kg of sample) = (OD of sample − OD of blank sample) × 5.2(1)

### 2.6. Statistical Analysis

All results of the present study were analyzed by t-tests using the least significant difference procedure of the SAS package program (release. 9.1.3 version, 2005). Significant differences were accepted if *p* < 0.05.

## 3. Results and Discussion

### 3.1. Growth Performance

Table 2 shows the effect of supplementation with RPMT on the growth performance of late fattening Hanwoo steers. Initial body weight was similar between the treatment groups, and final body weight was slightly but not significantly higher in the treatment group than in the control group. ADG was 34% higher in the treatment group than in the control group, but there was no statistical significance. The DMI was similar between the treatment groups, but the FCR was lower in the treatment group compared to the control group (*p* < 0.05).

Metabolism of restricted amino acids can affect the growth and normal metabolism of livestock, especially in beef cattle. ADG and energy availability may vary depending on the type or content of amino acids supplied from the small intestine [21]. Moreover, methionine is classified as a restricted amino acid in beef cattle and plays a pivotal role in cell metabolism and protein synthesis [5]. Wright and Loerch [22] reported that the addition of rumen-protected methionine and lysine improved the ADG and feed efficiency in beef cattle, and Park [6] also reported similar improvements to the present study in that the ADG of Hanwoo steers was improved due to the supplementation of amino acid-enriched rumen-protected fatty acids.

In addition, α-tocopherol (vitamin E) is known to prevent cell oxidation from free radicals [23], with improved ADG [11] and feed efficiency [24]. Hill and Williams [25] reported that 100 to 200 IU of vitamin E supplementation could improve the ADG of steers, especially in stressful situations. Thus, the results of previous studies support the results from this study.

### 3.2. Carcass Characteristics

Table 3 shows the effects of RPMT on carcass characteristics of Hanwoo steers.

The carcass weight and rib eye area were slightly but not significantly higher in the treatment group than in the control group. Backfat thickness was slightly lower in the control group than in the treatment group, but there was no significant difference. The marbling score tended to be increased by 24.1% in the treatment group compared to the control group; however, there was no significant difference. Between the treatment group showed similar results at the meat color, fat color, texture, and maturity. The meat quality grade score was slightly but not significantly higher in the treatment group than in the control group.

In this study, carcass weight and rib eye area tended to be higher in the treatment group with higher live weight than in the control group. According to the study by Lee et al. [26], carcass weight and rib eye area were higher in the group with the highest live weight among 11,601 Hanwoo steers; however, backfat thickness was high and the meat yield index and yield grade were low, which is in agreement with the results from the present study. In addition, because backfat thickness is the highest correlated with the decrease in carcass yield grade [27], decrease in the yield grade score of the treatment group is related to higher backfat thickness in the present study. On the other hand, Kim et al. [28] and Park et al. [29] reported that marbling scores were increased by the supplementation of rumen-protected methionine and lysine in Hanwoo steers, suggesting that methionine is a methyl donor for the transmethylation reaction [30] in lipid transport and biosynthesis. Although no statistical significance was observed in this study, the marbling score was increased by supplementation with rumen-protected methionine, which is similar to the results from previous studies.

In addition, the results of the present study were similar to previous studies in which α-tocopherol supplementation resulted in increased marbling score [31] and backfat thickness [32]. However, in some studies [24,33], there were no differences in the carcass yield and quality characteristics according to α-tocopherol supplementation. The difference in results is consider to be according to the difference in the period or level of α-tocopherol supplementation

### 3.3. Chemical Composition and Quality of the Longissimus Muscle

The effects of RPMT on the physicochemical characteristics in the longissimus muscle of Hanwoo steers are shown in Table 4.

The ether extract content of the longissimus muscle was slightly but not significantly higher in the treatment group than in the control group. Crude protein and ash were similar between treatments. Supplementation of RPMT did not affect the WHC. Shear force was slightly lower in the treatment group than in the control group, but no significant difference was observed.

The physicochemical characteristics in the longissimus muscle are closely related to the marbling score and meat quality grades in Hanwoo [34], and the moisture and ether extract contents of longissimus muscle were influenced by the proportion of marbling [35,36]. In addition, higher marbling (intramuscular fat deposition) decreases the shear force and cooking loss [37,38]. The results of the present study are similar to previous results in that higher marbling resulted in an increase in the ether extract and a decrease in cooking loss and shear force. Therefore, the results of the present study indicate that supplementation of RPMT may have some influence on the physicochemical characteristics in the longissimus muscle of Hanwoo steers.

The effects of RPMT on the fatty acid composition in the longissimus muscle of Hanwoo steers are shown in Table 5.

Supplementation of RPMT had little effect on the composition of individual fatty acids, saturated fatty acids, and monounsaturated fatty acids. Some polyunsaturated fatty acid ratios were slightly but not significantly higher in the treatment group than in the control group.

Fatty acid composition of meat can be influenced by the type of feed, fattening period, and meat quality grade [39]; in particular, fatty acid composition of the feed is the most important factor [40,41]. In the present study, the fatty acid composition between the treatments were similar, indicating that the supplementation of RPMT had little effect on the fatty acid composition of the longissimus muscle in Hanwoo steers.

Kim et al. [28] reported that fatty acid composition in the longissimus muscle was similar between treatments based on amino acid-enriched ruminally protected fatty acid levels, which is similar to the results of the present study. However, in the case of α-tocopherol, a strong antioxidative effect may help increase the unsaturated fatty acid ratio in the longissimus muscle by preventing the oxidation of unsaturated fatty acids in the cell membrane [9]; therefore, further investigation is needed in the future.

Table 6 shows the effect of supplementation with RPMT on CIE color and myoglobin composition in the longissimus muscle of Hanwoo steers during the storage period.

The ratio of DeoxyMb in the longissimus muscle increased with the storage period, whereas the OxyMb ratio tended to be decreased, regardless of the treatments. MetMb ratio was significantly lower in the treatment group than in the control group on day 9 of storage (*p* < 0.05). Supplementation with RPMT did not affect the brightness of the longissimus muscle during storage. The redness of the longissimus muscle was similar between treatments until day 6 of storage; however, it was higher in the treatment group than in the control group on day 9 of storage (*p* < 0.01).

Myoglobin is oxidized to DeoxyMb, OxyMb, and MetMb [42], and oxidation of pigment proteins has an adverse effect on meat color in the longissimus muscle [43]. The ratio of MetMb to the final oxidized form was also increased in the present study. The results of the present study are also in agreement with those of a previous study [44], which found that the MetMb ratio was increased with the storage period. In the present study, rumen-protected α-tocopherol resulted in a decrease of the MetMb ratio and an increase in redness on day 9 of storage. The result of this study was supported by the results of previous studies [45,46], which found that α-tocopherol, a potent antioxidant, has proved its effectiveness in preserving meat color. Lee et al. [47] reported a result similar to the present study that vitamin E supplementation resulted in delayed discoloration and decreased MetMb production in the longissimus muscle of Hanwoo steers.

Table 7 shows the effect of supplementation with RPMT on pH and TBARS in the longissimus muscle of Hanwoo steers during the storage period.

There was no significant difference between the treatments in the pH of longissimus muscle during the entire storage period, with the pH rapidly increased at 9th day of storage. The TBARS value was increased as the storage period was increased in all treatments, and the TBARS value on day 9 of storage was slightly but not significantly lower in the treatment group than in the control group.

pH, which is an important measure in meat quality assessment, affects meat color, hardness, rancidity, and water holding capacity [48]. Normal beef pH range is less than 5.75 [49]. In general, the pH of meat is reduced by the production of lactic acid during the early stages of storage and is gradually increased by the production of alkaline substances by proteolytic enzymes [50]. Similar to a previous study, a significant degree of proteolysis in the longissimus muscle in proportion to the storage period led to an increase in pH in the present study. In addition, we consider that supplementation with rumen-protected methionine and α-tocopherol has little effect on the pH in the longissimus muscle of Hanwoo steers.

TBARS is a measure of the level of malondialdehyde induced by lipid oxidation [51], which is influenced by fatty acid composition in the longissimus muscle. In particular, when the degree of unsaturation is high, lipid oxidation is promoted [52]. In the present study, although the proportion of PUFA was higher in the treatment group than in the control group (Table 4), the TBARS value tended to be lower in the treatment group as the storage period was increased; this is probably due to the antioxidant effect of α-tocopherol. α-tocopherol has been reported to be absorbed in the small intestine and then into tissues via the transport protein [46] and is known to accumulate effectively in the carcass [53]. In addition, the result of the present study was supported by previous studies [54,55], which found that the TBARS level of the longissimus muscle was decreased by supplementation with α-tocopherol.

## 4. Conclusions

The stress that occurs during the high temperature season reduces feed intake and decreases productivity in fattening beef cattle. In this study, the addition of RPMT did not have the effect on feed intake, but it improved ADG and FCR of Hanwoo steers in a heat stress environment and maintained CIE color stability of longissimus muscle. Therefore, supplementation of RPMT can have a favorable effect on the performance characteristics of beef cattle and help prevent oxidation of carcasses. However, since the group size was small in this study, in order to more clearly prove the effect of using RPMT, a study that increased the group size (= *n*) and amount added is considered necessary in the future.

## Figures and Tables

**Table 1:** Chemical composition of experimental diets (dry matter (DM) basis).

Items	Concentrate	Rice Straw
Dry matter (%)	90.52 ± 0.27	91.12 ± 0.54
Crude protein (%)	12.59 ± 0.11	4.76 ± 0.32
Ether extract (%)	4.02 ± 0.25	1.26 ± 0.08
Crude ash (%)	6.85 ± 0.10	7.98 ± 0.21
Crude fiber (%)	9.08 ± 0.51	33.25 ± 0.69
NDF ^1^ (%)	31.29 ± 0.36	69.80 ± 0.54
ADF ^2^ (%)	13.12 ± 0.48	40.91 ± 0.33

^1^ NDF, neutral detergent fiber; ^2^ ADF, acid detergent fiber.

**Table 2:** Effect of rumen protected methionine and α-tocopherol on growth performance of Hanwoo steers.

Items	Control	Treatment	Pr > |t|
Body weight (kg)			
Initial BW ^1^	714.00 ± 97.89	712.57 ± 78.37	0.98
Final BW	776.43 ± 104.83	795.86 ± 79.74	0.70
Total gain	62.43 ± 24.20	83.29 ± 15.21	0.08
Average daily gain	0.50 ± 0.20	0.67 ± 0.12	0.08
Feed intake (DM ^2^ kg/day/head)	9.92 ± 0.04	9.81 ± 0.04	0.12
Concentrate	8.72 ± 0.08	8.58 ± 0.07	0.15
Rice straw	1.20 ± 0.03	1.23 ± 0.03	0.07
Feed conversion ratio	22.55 ± 9.39 ^a^	15.09 ± 3.17 ^b^	0.04

^1^ BW, body weight; ^2^ DM, dry matter, ^a,b^ Means with difference superscript in the same row are significantly different (*p* < 0.05).

**Table 3:** Effect of rumen protected methionine and α-tocopherol on carcass characteristics of Hanwoo steers.

Items	Control	Treatment	Pr > |t|
Yield traits ^1^			
Carcass weight (kg)	446.29 ± 66.07	473.57 ± 62.39	0.44
Rib eye area (cm^2^)	73.86 ± 7.47	76.57 ± 11.30	0.61
Back fat thickness (mm)	12.14 ± 5.18	14.14 ± 4.91	0.47
Yield index	62.71 ± 4.64	61.16 ± 2.86	0.46
Yield grade (A:B:C, %)	14:43:43	0:43:57	-
Yield grade score ^2^	2.00 ± 0.82	1.43 ± 0.53	0.15
Quality traits ^3^			
Marbling score	3.57 ± 1.40	4.43 ± 0.79	0.19
Meat color	3.00 ± 0.00	3.00 ± 0.00	-
Fat color	4.00 ± 0.00	4.00 ± 0.00	-
Texture	1.14 ± 0.38	1.00 ± 0.00	0.36
Maturity	2.86 ± 0.38	2.86 ± 0.38	-
Quality grade (1^+^:1:2, %)	14:43:43	14:86:0	-
Quality grade score ^4^	2.71 ± 0.76	3.14 ± 0.38	0.21
Auction price (won/kg)	16,605 ± 2427	18,015 ± 780	0.19

^1^ Area was measured from longissimus muscle taken at 13th rib and back fat thickness was also measured at 13th rib; Yield index was calculated using the following equation: (68.184 − (0.625 × back fat thickness [mm]) + (0.130 × rib eye area [cm^2^]) − (0.024 × dressed weight amount [kg])) + 3.23; ^2^ A grade = 3, B grade = 2, C grade = 1; ^3^ Grading ranges are 1 to 9 for marbling score with higher numbers for better quality (1 = devoid, 9 = abundant); meat color (1 = bright red, 7 = dark red); texture (1 = soft, 3 = firm); maturity (1 = youthful, 9 = mature); ^4^ 1^+^ grade = 4, 1 grade = 3, 2 grade = 2.

**Table 4:** Effects of rumen protected methionine and α-tocopherol on physicochemical characteristics in longissimus muscle of Hanwoo steers.

Item	Control	Treatment	Pr > |t|
Moisture (%)	67.10 ± 3.63	64.60 ± 3.43	0.21
Crude fat (%)	12.38 ± 3.00	14.77 ± 3.98	0.23
Crude protein (%)	19.53 ± 2.63	19.65 ± 1.61	0.92
Crude ash (%)	0.99 ± 0.00	0.97 ± 0.04	0.39
WHC ^1^ (%)	76.39 ± 1.99	75.16 ± 2.64	0.34
Shear force (kgf)	5.68 ± 1.03	4.96 ± 0.67	0.15

^1^ WHC, water holding capacity.

**Table 5:** Effects of rumen protected methionine and α-tocopherol on fatty acid composition in longissimus muscle of Hanwoo steers.

Item	Control	Treatment	Pr >|t|
C14:0 (Myristic, %)	3.40 ± 0.37	3.51 ± 0.47	0.64
C16:0 (Palmitic, %)	28.43 ± 1.32	28.34 ± 1.56	0.91
C16:1n7 (Palmitoleic, %)	5.31 ± 0.79	5.99 ± 0.81	0.14
C18:0 (Stearic, %)	9.72 ± 0.83	10.04 ± 1.82	0.68
C18:1n9 (Oleic, %)	50.48 ± 1.60	49.19 ± 3.01	0.34
C18:2n6 (Linoleic, %)	2.35 ± 0.32	2.56 ± 0.38	0.27
C18:3n3 (α-linolenic, %)	0.23 ± 0.07	0.28 ± 0.02	0.13
C20:4n6 (Arachidonic, %)	0.04 ± 0.01	0.07 ± 0.04	0.19
C20:5n3 (Eicosapentaenoic, %)	0.03 ± 0.02	0.03 ± 0.01	0.74
SFA ^1^	41.55 ± 1.70	41.89 ± 2.90	0.79
MUFA ^2^	55.79 ± 1.60	55.17 ± 3.17	0.66
PUFA ^3^	2.65 ± 0.34	2.93 ± 0.41	0.19
*n*-3	0.26 ± 0.07	0.31 ± 0.03	0.17
*n*-6	2.39 ± 0.31	2.63 ± 0.40	0.25
*n*-6/*n*-3	9.47 ± 1.89	8.65 ± 1.43	0.38

^1^ SFA, saturated fatty acid; ^2^ MUFA, mono-unsaturated fatty acid; ^3^ PUFA, poly-unsaturated fatty acid.

**Table 6:** Effects of rumen protected methionine and α-tocopherol on myoglobin forms and Commission Internationale de l’Eclairage (CIE) color values in longissimus muscle of Hanwoo steers during refrigerated storage.

Item	Storage Days	Control	Treatment	Pr >|t|
DeoxyMb ^1^ (%)	0	19.98 ± 3.57	18.82 ± 2.28	0.48
	3	17.50 ± 3.42	17.96 ± 5.34	0.85
	6	27.91 ± 1.29	27.82 ± 1.05	0.89
	9	29.56 ± 6.98	28.33 ± 8.74	0.78
OxyMb ^2^ (%)	0	56.86 ± 6.07	57.37 ± 7.05	0.89
	3	47.57 ± 6.72	47.21 ± 7.80	0.93
	6	34.77 ± 2.72	35.97 ± 1.73	0.35
	9	16.92 ± 9.21	21.93 ± 10.13	0.35
MetMb ^3^ (%)	0	23.15 ± 3.30	23.81 ± 5.94	0.80
	3	34.93 ± 3.90	34.83 ± 4.50	0.96
	6	37.32 ± 1.66	36.20 ± 1.39	0.20
	9	53.52 ± 3.75 ^a^	49.74 ± 3.06 ^b^	0.04
Lightness (L*)	0	39.25 ± 2.85	38.11 ± 3.50	0.52
	3	38.22 ± 4.20	38.02 ± 2.39	0.82
	6	36.33 ± 1.57	37.74 ± 3.69	0.20
	9	37.78 ± 1.55	37.55 ± 1.75	0.83
Redness (a*)	0	24.44 ± 2.33	24.23 ± 1.75	0.70
	3	23.88 ± 1.50	23.54 ± 1.41	0.54
	6	22.99 ± 0.85	22.39 ± 2.32	0.40
	9	15.55 ± 2.29 ^b^	19.33 ± 2.63 ^a^	0.01
Yellowness (b*)	0	13.36 ± 0.77	13.44 ± 1.43	0.90
	3	12.52 ± 0.54	12.31 ± 1.38	0.72
	6	13.15 ± 1.38	12.81 ± 0.98	0.60
	9	10.14 ± 1.01	10.48 ± 2.18	0.72

^1^ DeoxyMb, deoxymyoglobin; ^2^ OxyMb, oxymyoglobin; ^3^ MetMb, metmyoglobin. ^a,b^ Means with difference superscript in the same row are significantly different (*p* < 0.05).

**Table 7:** Effects of rumen protected methionine and α-tocopherol on pH and TBARS values in longissimus muscle of Hanwoo steers during refrigerated storage.

Items	Storage Days	Control	Treatment	Pr >|t|
pH	0	5.41 ± 0.04	5.42 ± 0.06	0.82
	3	5.42 ± 0.04	5.44 ± 0.05	0.36
	6	5.45 ± 0.04	5.46 ± 0.06	0.81
	9	5.66 ± 0.11	5.68 ± 0.21	0.82
TBARS ^1^ (mg MDA/kg)	0	0.35 ± 0.04	0.38 ± 0.06	0.47
	3	0.42 ± 0.12	0.38 ± 0.18	0.62
	6	0.50 ± 0.11	0.47 ± 0.05	0.64
	9	0.57 ± 0.26	0.40 ± 0.14	0.12

^1^ TBARS, 2—Thiobarbituric acid reactive substances.
